# Comparing Anterior Versus Posterior Size Referencing in Patients Undergoing Simultaneous Bilateral Total Knee Arthroplasty: One Technique Per Knee

**DOI:** 10.1007/s43465-026-01690-9

**Published:** 2026-01-16

**Authors:** Sachin R. Tapasvi, Madhav Chowdhry, Anshu Shekhar, Komal S. Tapasvi, Matthew V. Dipane, Edward J. McPherson

**Affiliations:** 1 Department of Arthroplasty and Arthroscopy, The Orthopaedic Specialty Clinic, Pune, India; 2 Department of Orthopaedic Surgery, Jawaharlal Nehru Medical College, AMU, Aligarh, India; 3 Department of Orthopaedic Surgery, Sancheti Institute for Orthopaedics and Rehabilitation, Pune, Maharashtra India; 4 Department of Orthopaedic Surgery, David Geffen School of Medicine, UCLA, Los Angeles, USA; 5 Department of Orthopaedic Surgery, David Geffen School of Medicine, UCLA, Los Angeles, USA

**Keywords:** Anterior referencing, Posterior referencing, Total knee arthroplasty (TKA), Femoral sizing, Surgical technique, Measured resection

## Abstract

**Background:**

The preferred technique for femoral implant sizing in primary total knee arthroplasty (TKA) is debatable. Sizing is based on the anterior femoral cortex or posterior condylar reference. Using a single knee system, this study compared anterior referencing (AR) versus posterior referencing (PR) in patients undergoing simultaneous bilateral TKA, where one reference technique was randomized to each knee.

**Methods:**

This prospective study compared the two sizing references using one posterior stabilized knee system. The study included 81 subjects with osteoarthritis and similar varus deformity in both knees. All subjects underwent identical surgical procedures, aside from the selected femoral sizing reference. Subjects were followed for at least 2 years.

**Results:**

The two sizing techniques did not significantly differ in all measured radiographic, operative, and clinical parameters. The mean posterior condylar offset ratios were similar. Two-year mean knee flexion and Revised Oxford Knee Scores showed no difference. Eighty-four percent of patients stated no preference for either technique.

**Conclusion:**

Knees sized with anterior referencing had similar functional outcomes to those sized with posterior referencing. Using a current knee system with multiple sizing options, there is no discernable difference in all measured study parameters. Either reference is acceptable.

*Level of Evidence* 2 (Randomized cohort study).

## Introduction

Primary total knee arthroplasty (TKA) for degenerative gonarthrosis provides improvement in pain and function, with utilization rising globally [1–3]. Prosthetic implantation is a multistep process, combining technical execution with surgeon insight to optimize alignment and balance. Surgical techniques regarding end bone cuts and knee balancing are varied and continually debated [4–6].

One crucial step is the sizing and positioning of the femoral component in the sagittal plane, for which there are two techniques: anterior referencing (AR) and posterior referencing (PR). The preferred reference remains debatable and clinical comparison is challenging [7–9]. Patients have unique knee anthropometrics and undergo individualized degenerative trajectories creating specific deformity [10–13]. Soft tissue compliance between patients also differs significantly. To compare differing references, it would be best to compare knees of similar anatomy, deformity, and soft tissue compliance. This could be accomplished by comparing knees in a single patient.

This study was designed to assess just such a comparison. We performed a prospective, randomized study comparing AR to PR in patients undergoing simultaneous bilateral TKAs. One reference technique was performed in each knee, while all other steps remained unchanged. We hypothesize that PR knees will have better overall function because the posterior flexion gap will be consistently reproduced.

## Materials and Methods

This study received IRB approval prior to initiation. Subjects underwent bilateral simultaneous TKAs using identical surgical technique, other than the randomized femoral sizing. Subjects were consented and enrolled between May 2019 and July 2022. Inclusion criteria were osteoarthritis (OA) with failed non-operative treatment and a mechanical varus alignment within 5° of the contralateral knee. Exclusion criteria included valgus knee deformity, flexion contracture > 15°, extra-articular knee deformity, flexion < 90°, and inflammatory or traumatic arthritis. The knee assigned to each technique was selected by simple randomization with a 1:1 allocation ratio using a random numbers generator performed by a research fellow upon approval of patient enrollment and the envelop sealed. The first side of the bilateral procedure was selected by the patient. The surgeon was informed of knee randomization just prior to surgical start by opening of the envelope.

Pre-operative radiographs included digitized anterior–posterior (AP), lateral, patellofemoral, and full-length standing AP views. Radiographic measurements included limb alignment defined by the mechanical Hip-Knee-Ankle Angle (mHKA) and the medial Proximal Tibial Angle (MPTA) [14]. Varus limb alignment was defined as an mHKA angle < 180°. Tibial vara was defined as an MPTA angle < 90°. These measurements ensured knee deformity matching. The preoperative bony posterior condylar offset ratio (PCOR) on lateral radiographs was measured and compared to 3-month radiographs [15]. All measurements were made using a digital software tool (Image J 1.52a, National Institute of Health, Bethesda, USA).

### Surgical Protocol

Anesthesia was standardized, using an epidural with a single shot pre-surgical adductor canal block. A second generation cephalosporin (Cefuroxime 1.5 g) was administered perioperatively for 24 h. The pneumatic tourniquet was inflated before skin incision and deflated after cement setting. A posterior-stabilized, fixed-bearing design was used in all cases (Attune® Knee System, DePuy, Warsaw, USA). Implants were cemented using Palacos® R bone cement (Heraeus Medical GmbH, Hanau, Germany), and all patellae were resurfaced.

Surgeries were performed by the first author (SRT) with manual instrumentation, utilizing a measured resection technique. An anterior-medial subperiosteal tibial dissection was performed, followed by release of the deep medial collateral ligament. All osteophytes were removed. Distal femoral resection was executed with a distal valgus cut angle of 5°. The proximal tibia was resected perpendicular to the tibial mechanical axis with a 3° posterior slope. Femoral and tibial condylar surface thicknesses were measured with a digital caliper.

The femoral rotation line was marked on the distal femur with a jig and sterile pen, using the Anterior–Posterior Axis Line (APAL) as defined by Whiteside [4, 16, 17]. The transepicondylar axis line (TEAL), as described by Berger et al., was next marked [18, 19]. The rotation of the APAL and TEAL was compared to assure concordance.

**Fig. 1:**
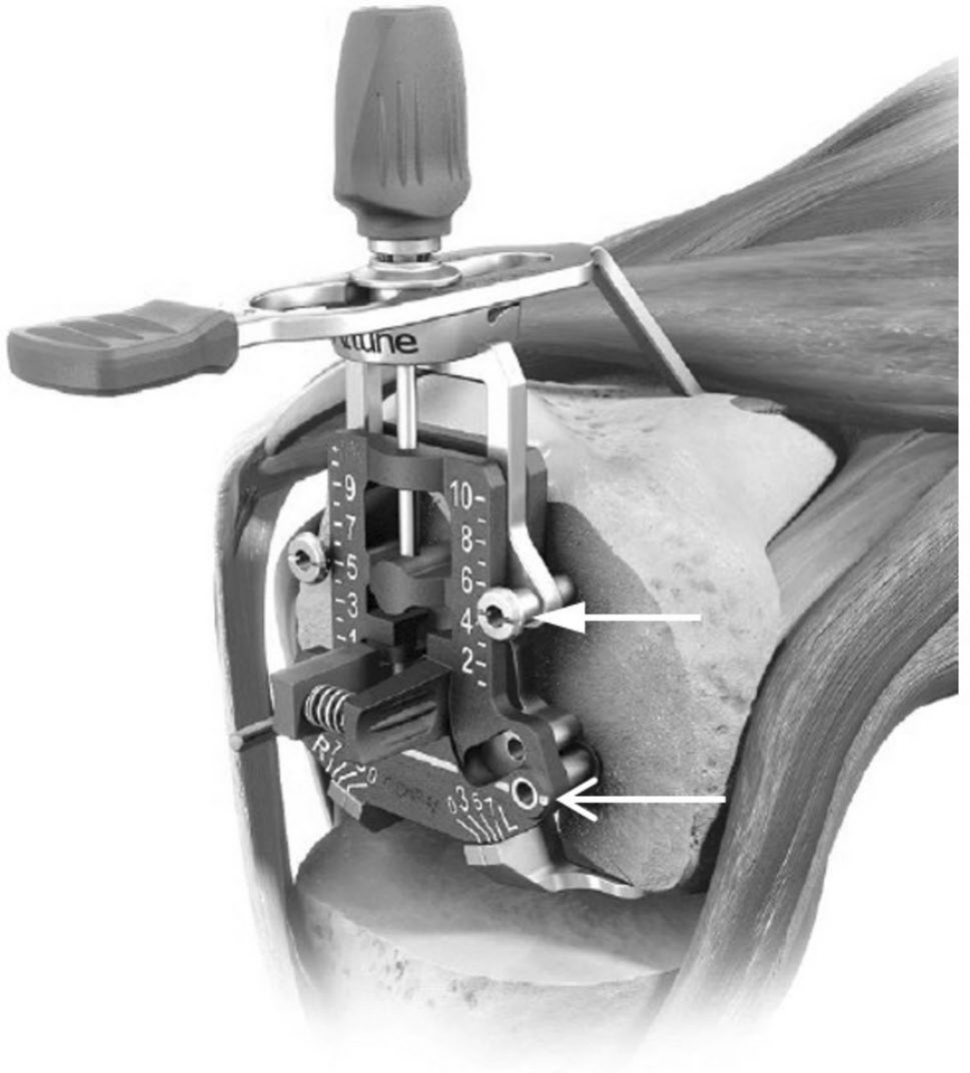
Illustration of sizing/rotation guide used in this study. The jig allows for both anterior and posterior referencing. With anterior referencing, the upper holes are pinned first (solid arrow), locking the upper tower and fixing the anterior reference stylus in place. This allows the posterior jig section to slide. The surgeon then selects the posterior position and chooses the femoral size. With posterior referencing, the lower holes are pinned first (open arrow), locking the lower section and fixing the posterior reference (metallic foot pads). The upper tower is allowed to slide. The surgeon then selects the anterior position and chooses the femoral size. Illustration adapted from DePuy Intuition Instrument Technique Guide (DePuy, Warsaw, USA)

Femoral component sizing was determined using the randomized reference technique. DePuy Intuition Instrumentation (DePuy, Warsaw, USA) was utilized for both techniques. The sizing jig is illustrated in Fig. 1**.** For AR, the jig was placed flush against the distal femur with the metallic condylar pads beneath each posterior femoral condyle. Rotation was established and the anterior cortical reference identified with the stylus, which was then locked. Drill holes and pins for the anterior reference were placed. These pins locked the anterior tower, allowing the lower jig apparatus to slide. The lower sizer section was positioned to select the posterior condylar cut based on surgeon selection, to come as close to measured resection as possible. The selected 4-in-1 cutting block was placed. Anterior, posterior, and chamfer cuts were made before the box cut.

For PR, the jig was placed identical to the method described above. Rotation was established and holes and pins drilled for the posterior reference. These pins locked the posterior jig section, allowing the anterior tower apparatus with the cortical stylus to slide. The upper sizer section was positioned to select the anterior cortical cut based on surgeon selection to come as close to measured resection as possible. The sizing stylus was placed onto the lowest portion of the anterior cortical bone within the middle 1/3 cortex. The selected 4-in-1 cutting block was placed and the anterior, posterior, and chamfer cuts made before the box cut.

Sizing was based on the following algorithm. The Attune had 10 femoral AP sizes with each size growing symmetrically in 3 mm increments. Therefore, when exactly in-between sizes, the maximal error will be 1.5 mm assuming symmetrical anterior and posterior position. Full squatting was top priority, and the patellofemoral gap was never overstuffed. When using anterior referencing and when in-between sizes, the sizing rule was selecting the femoral size with the smaller gap discrepancy and then fine-tuning sagittal balance. For posterior referencing, the posterior reference pads positioned implant condyles in the native position; the only decision in size was how much anterior bone to remove, never overstuffing the patellofemoral gap. A partial notch (≤ Tayside 2) was allowed to achieve our sizing goals.

Balancing was performed with trial implants. Releases were performed as needed to achieve full extension. Flexion balancing was assessed by lifting the thigh and allowing flexion by gravity, with a goal of ≥ 120°. Coronal balancing was assessed with stress testing at full extension, 60°, and 90°. Tibial implant rotation was set with a “tibial float technique,” using a metallic femoral trial and an articulating tibial trial without a keel [20–22]. Tibial rotation was marked where the bearing mated congruently through the majority of knee range. Once marked, the keel was prepared, and final balancing was rechecked and adjusted. Knees were closed in flexion of 90° without a drain. Subjects began therapy that afternoon or the following morning.

Assessment scores were performed at 3 months, 6 months, 1 year, and 2 years. All medical complications and reoperations were recorded. Function was assessed by range of motion and Revised Oxford Knee Score (ROKS) [23–27]. Radiographs were conducted at 3 months and annually. Data analysis was performed using Excel Software (Microsoft, Redmond, USA). *χ*^2^ testing was performed to compare categorical variables. Two-tailed paired *t* testing with equal variance was performed for statistical significance of numerical variables between categories at *P* < 0.05.

## Results

Enrollment totaled 85 subjects. Four were excluded: two required medullary stem support with medial augmentation in one knee, necessitating a knee system change and invalidating comparable techniques, one required a constrained high post bearing, again necessitating a system change, and one underwent revision surgery for aseptic loosening after 8 months. Eighty-one subjects completed the minimum 2-year follow-up for study inclusion. There were 63 women (77.8%) and 18 men (22.2%). The mean age was 66.1 years (range 49–79). Tourniquet time averaged 49.27 ± 6.6 min for AR knees and 49.23 ± 4.5 min for PR knees (*P* = 0.96). Given unequal variances, a Welch’s *t* test compared mean tourniquet times. The difference remained nonsignificant (*t* = 0.045, *P* = 0.964). The number of lateral retinacular releases (LRR) was similar, with three (3.7%) in AR knees and two (2.5%) in PR knees (*P* = 0.206). Using the Tayside classification, there were three cortical notches (two grade 1’s, one grade 2) in AR knees and four (all grade 1’s) in PR knees (*P* = 0.206) [9, 28].

### Bone Resection Measurements

Bone cut measurements are summarized in Table 1. The mean values of distal femoral and proximal tibial cuts were similar. There were no statistical differences between the two techniques in any bone resection measurements.

**Table 1:** Thickness of resected bone cuts from the femur and tibia

Site	Side	Rotation axis		*P* value
		AR (mm)	PR (mm)	
Distal femur	Medial	8.1 ± 1.3	7.9 ± 1.2	0.42
	Lateral	8.6 ± 1.0	8.5 ± 1.2	0.30
Proximal tibia	Medial	1.1 ± 0.7	1.1 ± 0.7	0.12
	Lateral	7.9 ± 2.0	7.7 ± 2.2	0.52
Posterior femur	Medial	9.7 ± 1.2	9.7 ± 0.9	0.94
	Lateral	7.1 ± 1.43	6.9 ± 0.9	0.29
Anterior femur	Medial	5.5 ± 2.0	5.6 ± 2.3	0.79
	Lateral	9.9 ± 2.2	10.0 ± 2.3	0.90

### Implant Size Variation

Implant size differences were not statistically significant and are summarized in Table 2. The femoral implant was identical in both knees for 49 subjects (60.5%), smaller in the PR knee for 19 subjects (23.5%), and larger for 13 subjects (16%) (*P* = 0.32). Tibial implant size was identical for 67 subjects (82.7%), smaller in the PR knee for 10 subjects (12.3%), and larger for 4 subjects (4.9%) (*P* = 0.33). Mean polyethylene (PE) thickness was similar, averaging 5.86 mm (range, 5–12) in AR knees and 5.81 mm (range, 5–10) in PR knees (*P* = 0.80). PE thickness was equal in 42 subjects (51.8%), thinner in the PR knee for 23 subjects (28.3%), and thicker for 16 subjects (19.7%) (*P* = 0.81).

**Table 2:** Variation in implant sizes in the same patient

Femur implant	Number	Tibia implant	Number	Polyethylene insert	Number
AR > PR	19	AR > PR	10	AR > PR	23
AR = PR	49	AR = PR	67	AR = PR	42
AR < PR	13	AR < PR	4	AR < PR	16

### Posterior Condylar Offset

The posterior condylar offset ratio (PCOR) was comparable between groups **(**Table 3**)**. Postoperative mean PCOR was 0.47 (range, 0.32–0.59) in AR knees and 0.46 (range, 0.32–0.57) in PR knees (*P* = 0.78). Compared to preoperative PCOR, postoperative AR PCOR was similar (± 10%) in 12 subjects (14.8%), larger (> 10%) in 39 subjects (48.1%) and smaller (> 10%) in 30 subjects (37.1%). PR PCOR was similar in 15 subjects (18.6%), larger in 35 subjects (43.2%), and smaller in 31 subjects (38.2%). The distribution pattern within both groups was similar (*P* = 0.75).

**Table 3:** Posterior condylar offset ratio (PCOR) measurements

	AR	PR	*P* value
Mean pre-operative PCOR	0.46 ± 0.05	0.46 ± 0.06	0.79
Mean post-operative PCOR	0.47 ± 0.05	0.46 ± 0.05	0.78
*P* value	0.26	0.79	

### Patient Outcomes

Functional outcomes are presented in Table 4. All measured parameters were similar in both groups. Mean extension and flexion ranges at 2 years were similar. Pre-operative ROKS were comparable. ROKS at 2 years were similar and improvements in ROKS were comparable. At last follow-up, subjects were asked for their preferred knee: 68 subjects (83.5%) stated no preference, 5 (6.1%) preferred the PR knee, and 8 (9.8%) preferred the AR knee.

**Table 4:** Functional outcome scores

Outcome	AR	PR	*P* value
Extension ROM 2-year follow-up	0.9 ± 1.8	0.8 ± 1.7	0.716
Flexion ROM 2-year follow-up	125.3 ± 5.7	124.4 ± 6.7	0.900
ROKS Pre-Op	17.5 ± 6.1	18.2 ± 7.1	0.51
ROKS 2-year follow-up	43.4 ± 3.8	44.2 ± 4.1	0.19
Improvement in ROKS	25.9 ± 7.2	26.0 ± 7.3	0.93

### Complications

The only reoperation was detailed above for aseptic tibial loosening. No knees required manipulation. There was one superficial wound infection after 14 days, requiring oral antibiotic with complete resolution. There were no supracondylar fractures or periprosthetic joint infections (PJI).

## Discussion

Femoral component sizing is a watershed moment in primary TKA and reference selection, either anterior or posterior, remains debatable. With AR, femoral implant size increases posteriorly from the anterior cortical reference, thus affecting the flexion gap. Conversely, with PR, femoral implant size increases anteriorly from the posterior condylar reference, affecting the patellofemoral gap [29]. The advantages and disadvantages of the two references are outlined in Table 5.

**Table 5:** Comparison of anterior referencing versus posterior referencing

	Anterior referencing	Posterior referencing
Advantages	Lower risk of femoral notch fracture	Lower risk of flexion gap errors
Disadvantages	1. Excess condylar bone removal with potential for flexion gap laxity	1. Excess trochlear bone removal creating a relevant notch
	2. Too little condylar bone removal with potential for flexion gap tightness	2. Too little bone removal with potential for patellofemoral gap tightness

This raises the question: does the reference selection significantly impact TKA function? Femoral implant sizes can closely match sagittal bone size, with implant increments differing as little as 2.0 mm. This contrasts with first-generation implants, where changes in sizing see differences as great as 5–7 mm [30]. Furthermore, polyethylene bearings are produced in 1 mm increments, allowing fine-tuning of bearing gaps. This is also true for patellar sizing, where standard and low-profile implants can closely match the native patellofemoral gap [29]. Another question is whether mis-sizing has greater consequences with a specific technique? Downstream effects of mis-sizing can be accommodated with knee balancing and bearing adjustments, but one technique may impart functional limitations. Objective comparison of TKA function between operative techniques is challenging. The true effect of the selected technique is best determined by keeping all surgical steps uniform and comparing knees of similar size, deformity, and soft tissue pliability. This is what our study aimed to accomplish.

This study demonstrated no significant differences across all measured parameters when comparing anterior and posterior size referencing. In all operative and radiographic measurements, perioperative events, and assessed clinical function, there were no statistical differences. We hypothesized that anterior referencing would more adversely affect knee function due to mismatch of the flexion gap, while posterior referencing would better recreate the flexion gap, translating to better function. Our results refuted this hypothesis.

Comparing both techniques, we noted no difference in mean anterior and posterior femoral bone resections or cortical notching. The similar rates suggest notching results from surgeon selection bias rather than forced sizing. Differences in PCOR between sides were not significant. Mean knee flexion at 2-year follow-up was no different, and ROKS were similar. Most subjects (60.4%) had equal femoral sizing; we believe differences in knee sizing were related to subtle surgeon selection bias rather than forced sizing. We acknowledge visual selection of femoral implant size is fraught with intra-observer variability. However, with all other parameters kept similar, this randomized study suggests that surgeon selected implant size via anterior or posterior referencing are equally acceptable techniques.

We explain our refuted hypothesis with several reasons. First, incremental femoral size changes were small. The Attune femur size grows symmetrically in 3mm increments. When using AR and exactly in-between sizes, the flexion discrepancy between the native femur and selected implant size is at most 1.5 mm, but usually smaller. This small discrepancy was not difficult to compensate with balancing techniques. Second, we were meticulous in testing flexion during trialing. We prioritized flexion gap stability to allow full squatting. We never left the flexion gap loose nor tight. During trialing, we performed a gravity “lift-up test” lifting the thigh with both thumbs to confirm full flexion by gravity. Second, we checked carefully for instability, with a mid-flexion instability stress test [31]. We also tested flexion instability using a “coronal shift test” performed at 90 degrees flexion. In this maneuver, a varus/valgus coronal translation stress is applied using a tibial trial insert without a central posterior stabilized (PS) post. Coronal subluxation indicates loose flexion tension requiring adjustment.

This study has several strengths. Foremost is its uniqueness in standardizing the many parameters in the TKA procedure to provide the best objective comparison of anterior-to-posterior size referencing. The selection criteria were strict, with comparable knee deformity and pliability paramount. Only one knee system was used. Employing a single surgeon kept surgical technique uniform and minimized size selection bias. The femoral sizing/rotation jig allowed for both referencing options and simple implementation.

However, with a highly selective study, there are limitations. By selecting OA candidates, our results cannot be extrapolated to other arthritic conditions where extemporaneous changes in surgical technique are required. For example, a posttraumatic knee with limited flexion and non-pliable tissues may benefit more from anterior referencing to increase the bony flexion gap to improve flexion. Similarly, these results cannot be espoused as a recommendation for valgus knee deformities. This is most important in knees with hypoplastic lateral femoral condyles, where posterior referencing more adversely affects rotation and sizing [32–34]. In this scenario, most surgeons agree that posterior condylar referencing is to be eschewed. We also note that our study had 3.5 times more women than men. This reflects the distribution ratio of osteoarthritis in India [35, 36]. In theory, anthropometric relations, including the landmarks used in this study, should not be significantly affected by sex, but we cannot definitively support this statement [37]. Females in this cultural region tend to have taller lateral trochlear profiles than males and may be more susceptible to lateral side notching [11, 13, 38]. Ideally, the study would have had an equal ratio. This study also contained four subject exclusions, three where one knee required additional augmentation/constraint, thus invalidating comparison between techniques. We selected subjects with comparable knee deformities, but this does not always confer symmetrical soft tissue integrity. For example, one knee may have a thrust, while the other does not. Increased laxity affects soft tissues and bone wear, requiring implant augmentation. We believe this difference in tissue integrity explains why one knee of these pairs required more support/constraint. In retrospect, we could have performed a gait test to ensure both knees were kinematically similar. We believe the fourth exclusion for aseptic tibial loosening was related to implant design and not to femoral referencing. Early tibial implant loosening was notable with the Attune tibia, a problem that has been subsequently rectified [39, 40]. We also acknowledge a relatively modest study size, illustrating the inherent challenges of strict selection criteria and subject recruitment. We note that, to our knowledge, this is the largest randomized comparison of the two techniques [8, 41–43]. Among patients undergoing simultaneous TKAs with comparable deformities and soft tissue pliability, it also represents the most rigorous comparative analysis currently available. We believe our study provides valuable information: by employing either referencing technique, acceptable sizing occurs with proper balancing.

## Conclusion

In patients with osteoarthritis and varus gonarthrosis, knees sized with anterior referencing had similar functional outcomes to those sized with posterior referencing. Using a current knee system that provides multiple implant sizing options, we found no discernable difference in all measured parameters. Both techniques are acceptable.

## Data Availability

All relevant patient data is provided within the manuscript.
